# Effect of *Amomum villosum* essential oil as an additive on the chemical composition, fermentation quality, and bacterial community of paper mulberry silage

**DOI:** 10.3389/fmicb.2022.951958

**Published:** 2022-07-22

**Authors:** Maoya Li, Xueying Fan, Qiming Cheng, Yulian Chen, Jianhua Long, Yao Lei, Ping Li, Chao Chen

**Affiliations:** ^1^College of Animal Science, Guizhou University, Guiyang, China; ^2^Key Laboratory of Animal Genetics, Breeding and Reproduction in the Plateau Mountainous Region, Ministry of Education, Guizhou University, Guiyang, China

**Keywords:** *Amomum villosum* essential oil, paper mulberry, silage, additives, fermentation quality, bacterial community

## Abstract

Paper mulberry (*Broussonetia papyrifera* L., PM) is being used as a new type of animal protein feed to address the feed crisis. To investigate the effect of additives on the chemical composition, fermentation quality, and bacterial community of PM silage (at room temperature, 25°), paper mulberry was fermented with formic acid (FA), *Amomum villosum* essential oil (AVEO) and lactic acid bacteria (LAB) inoculant treatments. The results showed that fresh PM had a low water-soluble carbohydrate (WSC) content and large amounts of unclassified bacteria. Compared with the CK and LAB treatments, the FA and AVEO treatments significantly (*P* < 0.05) decreased the pH and increased the lactic acid content of PM silage after 60 days of ensiling. In the AVEO-treated silages the abundance of *Lactococcus* in the early stage of ensiling increased by 14.09%, the abundances of *Levilactobacillus* and *Lentilactobacillus* in the late stage of ensiling increased by 58.34 and 91.12%, respectively, and the abundance of *Stenotrophomonas* decreased by 94.71%, resulting in improved PM silage quality. These results confirmed that AVEO could potentially be developed as a new additive for improving the fermentation quality of silage.

## Introduction

With the rapid development of China’s livestock industry, traditional feed is no longer sufficient to fulfill the needs of livestock (Dong et al., 2020). The exploitation of new feed resources to alleviate the feed crisis has been demonstrated (Guo et al., 2021). Paper mulberry (*Broussonetia papyifera* L., PM) is a perennial tree or shrub that is highly adaptable, fast growing, affordable to produce, and widely distributed (Peñailillo et al., 2016). China has cultivated approximately 300,000 hectares of PM as animal feed because of its high crude protein (CP) content and its high levels of vitamins and amino acids (Liu et al., 2019). Moreover, some studies have indicated that feeding with PM silage could enhance the immunity and antioxidant functions of beef cattle and dairy cows (Hao et al., 2020; Tao et al., 2020).

PM is harvested in the high-temperature and high-precipitation season, causing PM to become moist, and ensiling has been indicated to be the best way to preserve PM (Zhang et al., 2019). However, Cheng et al. (2021) observed that untreated PM was difficult to ensile due to its high buffering capacity and due to the low lactic acid bacteria (LAB) concentration (< 10^5^ cfu/g FM) in fresh forage. LAB additives are widely used to accelerate the ensiling process, limit the growth of harmful microorganisms, and improve the quality of PM silage (Cheng et al., 2021; Du et al., 2021; Wang et al., 2021). Since the adaptability and developmental processes of LAB in forage during ensiling are unknown, LAB additives do not always improve the fermentation quality of silage. Kobayashi et al. (2010) discovered that adding LAB additives derived from *Leymus chinensis* silage had no significant effect on the fermentation quality of PM silage, as indicated by the high pH value (> 6) and ammonia nitrogen (NH_3_–N) content (> 16% TN). Therefore, it is imperative to find LAB additives or other additives that can significantly improve the silage quality of PM.

Formic acid (FA) is commonly used as a chemical additive to rapidly reduce the pH of silage at an early stage of ensiling, suppress the growth of harmful microorganisms, and enhance silage quality (Wei et al., 2021). According to Andrade and Melotti (2004), adding 0.5% FA can improve the fermentation quality of elephant grass and increase animal digestibility. To date, there has been no report on the effect of FA on the quality of PM silage. Plant essential oils are naturally occurring secondary metabolites derived from aromatic plants that have a wide range of antimicrobial properties (Soycan-Önenç et al., 2015). Numerous studies have demonstrated that plant essential oils affect the growth and metabolism of a variety of microorganisms (rumen bacteria, gram-positive bacteria, and fungi) (Wallace, 2004). *Amomum villosum*, an herbaceous plant in the ginger family, is a famous traditional Chinese medicine (Chen et al., 2018). *A. villosum* essential oil (AVEO) is an edible spice oil that exhibits broad-spectrum antibacterial activity (Suo et al., 2018). It can be utilized as a food additive, animal feed supplement, or medicine (Hou and Jiang, 2013). Suo et al. (2018) found that AVEO significantly inhibited the growth of a range of harmful bacteria, including *Escherichia coli*, *Pseudomonas aeruginosa* and *Staphylococcus* sp. Moreover, Chen et al. (2018) showed that AVEO significantly inhibited lipopolysaccharide from entering blood circulation. This inhibitory effect may be associated with the beneficial effects on the equilibrium of the animal intestinal microflora (Chen et al., 2018). Although *A. villosum* has been widely used in the treatment of gastrointestinal diseases in animals, whether AVEO can be applied to improve PM silage quality and the underlying mechanisms of action are still unknown. The objective of our present study was to evaluate the effects of LAB, FA, and AVEO on the chemical composition, fermentation quality, and bacterial community of PM silage. Our hypothesis was that AVEO can improve the silage quality of PM.

## Materials and methods

### Silage preparation

Whole PM plants (*Broussonetia papyifera* L. Zhongke No.1) were harvested and chopped to a length of 1–2 cm on May 1st, 2021, in Changshun County, Guiyang city, Guizhou Province. To produce silage, approximately 300 g of chopped PM was mixed homogenously with each additive, packed manually into polyethylene bags (25 cm × 30 cm), and then vacuum packed using a vacuum packing machine (SJ-400, Shanghai Precision Machinery Manufacturing Co., Ltd.). The treatments were as follows: (1) CK (without additives), distilled water applied at 5 mL kg^–1^ fresh weight (FW); (2) FA, applied at 5 mL kg^–1^ FW (88%) (Zhongke Jiayi Biological Engineering Co., Ltd., Shandong, China); (3) LAB, combined application of *Lactiplantibacillus plantarum* and *Lactiplantibacillus buchneri* (Zhongke Jiayi Biological Engineering Co., Ltd., Shandong, China) at 2 × 10^7^ cfu/g FW; (4) AVEO, applied at 1 ml kg^–1^ FW (Baishengyuan Industrial Co., Ltd., Yang Jiang, China). Silages were stored at room temperature (22–25°C) and were opened in triplicate for each treatment after 3, 7, 15, 30, and 60 days of ensiling to analyze fermentation quality, after 3 and 60 days of ensiling to analyze microbial diversity and after 60 days of ensiling to analyze chemical composition.

### Analysis of chemical components and fermentation characteristics

Each sample was dried at 65°C, crushed, and sieved through a 0.5 mm sieve to estimate the DM content. Both the neutral detergent fiber (NDF) and acid detergent fiber (ADF) levels were analyzed using the methods of Van Soest et al. (1991). The water-soluble carbohydrate (WSC) content was determined by the anthrone colorimetric method as described by Zhang et al. (2017). The crude protein (CP) content was determined by the method of AOAC (1990).

Each 20 g silage sample was mixed homogeneously with 180 mL of sterile water for 3 min and then filtered through four layers of cheesecloth. The filtrate was used to measure the pH, organic acid content, and ammonia nitrogen (NH_3_-N) content. The concentrations of organic acids [lactic acid (LA), acetic acid (AA), propionic acid (PA), and butyric acid (BA)] were measured using high-performance liquid chromatography as described by Mu et al. (2021). The levels of organic acids were determined using an Agilent 1260 HPLC system with a 210 nm ultraviolet detector and an Acclaim TM Organic Acid column (Dionex Co., Ltd., Sunnyvale, CA, United States). The NH_3_-N content was measured as described by Broderick and Kang (1980) using the phenol–hypochlorite reaction.

### DNA extraction, amplification, and sequencing

To measure the bacterial community compositions of fresh and ensiled PM, each sample was immediately transported to Biomarker Technologies (Beijing, China). The bacterial community in the samples was analyzed by second-generation sequencing technology as described by Chen et al. (2020). The total DNA from each sample was extracted by a Power Soil DNA Isolation Kit (MO BIO Laboratories) according to the manufacturer’s protocol. After purification, the DNA was diluted to 1 ng/mL using sterile water. The 16S rDNA V3–V4 regions were amplified using a forward primer (50-ACTCCTACGGGAGGCAGCA-30) and reverse primer (50GGACTACHVGGGTWTCTAAT-30) combined with specific barcode sequences. The total PCR amplification volume was 50 μl, which included 10 μl of buffer, 0.2 μl of Q5 High-Fidelity DNA Polymerase, 10 μl of High GC Enhancer, 1 μl of dNTPs, 10 μM each primer, and 60 ng of genomic DNA. The polymerase chain reaction (PCR) was performed under the following conditions: initial activation of the hot-start polymerase at 95°C for 4 min, followed by 15 cycles of 95°C for 60 s, 60°C for 40 s, and 72°C for 60 s and a final extension at 72°C for 10 min. The final PCR products were pooled and quantified using the Quant-iT™ dsDNA HS Reagent. High-throughput sequencing was conducted on an Illumina HiSeq 2500 platform (2 × 250 paired ends) by Biomarker Technologies Corporation (Beijing, China) according to protocols described by Caporaso et al. (2012) and Kozich et al. (2013). Sequences with ≥ 97% comparability were assigned to the same operational classification unit (OTU) using UPARSE software. After standardizing the OTU abundance information, alpha diversity indexes (Chao, Shannon, Simpson, and Ace) and coverage values were computed using QIIME software (version 2.15.3).

### Statistical analyses

The chemical composition of PM mixed with additives was evaluated using one-way analysis of variance in SPSS Statistics (version 22.0) software (IBM Crop., Armonk, NY, United States). Data for fermentation quality and the bacterial community were analyzed *via* two-way analysis of variance to evaluate the effects of additives (T), ensiling period (D), and their interaction (T × D). The means were then compared to determine significance using Duncan’s multiple range method. All statistical analyses were performed using the general linear model procedure with SPSS 26 software (IBM Crop., Armonk, NY, United States). Significance was declared at *P* < 0.05 unless otherwise noted.

## Results and discussion

### Chemical compositions of fresh and ensiled paper mulberry

The chemical compositions of fresh and ensiled PM are shown in Table 1. The DM content of fresh PM was 28.87% FM, which was similar to the result of Hao et al. (2021), who reported that the DM content of whole PM plants was 26.6% FM. The NDF and ADF levels in PM were 66.70% DM and 39.92% DM, respectively, which were much higher than those observed by Du et al. (2021) (45.67% DM and 16.68% DM). The NDF and ADF levels observed in our study being higher because the previous study focused on PM leaves, while this study focused on the whole PM plant. However, the CP content of fresh PM was 15.67% DM, which was similar to that observed by Sun et al. (2022) (16.47% DM). The presence of sufficient WSC content (6–7% DM) in the fresh material is essential to ensure the quality of silage fermentation (Smith, 1962). However, the WSC content of fresh PM (2.07% DM) was < 6–7% DM in our study. As a result, silage additives were required to improve the fermentation quality of PM during ensiling (Dong et al., 2020).

**TABLE 1:** Chemical composition of fresh material and silage after 60 days of ensiling.

Items	Fresh forage	Silages				*P-value*
		CK	FA	LAB	AVEO	
DM%FM	28.87 ± 0.08	25.83 ± 0.44BC	28.77 ± 0.70A	24.22 ± 0.16C	26.48 ± 1.02B	0.008
CP%DM	15.67 ± 0.49	14.98 ± 0.78	16.35 ± 0.15	15.61 ± 0.22	15.51 ± 0.05	0.202
NDF%DM	66.70 ± 0.04	31.21 ± 0.54B	40.32 ± 1.16A	37.5 ± 0.91A	39.90 ± 2.03A	0.004
ADF%DM	39.91 ± 1.72	25.26 ± 0.73B	35.14 ± 1.07A	33.65 ± 1.00A	36.57 ± 1.56A	<0.001
WSC%DM	2.07 ± 0.04	0.71 ± 0.01B	1.34 ± 0.10A	1.19 ± 0.04A	1.16 ± 0.02A	<0.001
NH_3_-N%DM	-	2.44 ± 0.06AB	2.58 ± 0.01A	2.36 ± 0.07BC	2.23 ± 0.07C	0.018

*^A–C^Means of additive treatments within a row with different superscripts differed at 60 days of ensiling (P < 0.05). CK, without additives; FA, formic acid; LAB, Lactiplantibacillus plantarum combined with Lactiplantibacillus buchneri; AVEO, Amomum villosum essential oil; DM, dry matter; CP, crude protein; NDF, neutral detergent fiber; ADF, acid detergent fiber; WSC, water-soluble carbohydrate; NH_3_-N, ammonia nitrogen; FM, fresh matter; SEM, standard error of the mean.*

The additive significantly (*P* < 0.05) affected the DM, NDF, ADF, NH_3_-N, and WSC levels in the silage. The DM content is an important indicator in silage, as LAB require moisture for growth and reproduction (Xu et al., 2020). The DM content in all the PM silages was lower than that in fresh forage. According to Ogunade et al. (2016), this decrease could be due to the metabolism of soluble substrates by microbial fermentation. The DM content of the FA-treated silage was significantly higher than that of the other treated silages (*P* < 0.05). The addition of FA can effectively restrict the growth of harmful microorganisms, thereby minimizing DM loss (Wei et al., 2021). Moreover, the FA-, LAB-, and AVEO-treated silages had greater NDF and ADF levels than the CK silage (*P* < 0.05). However, consistent results were reported by Sun et al. (2022), who found that silage additives can increase the NDF and ADF levels in PM silage. This result might be attributed to the reduction in DM content (Li et al., 2021a). The WSC content in forage plays an essential role in the production of LA during ensiling. Compared with the CK, the additive treatment significantly (*P* < 0.05) increased the WSC content. Similar results were obtained by Li et al. (2021a), who reported that inoculation with additives increased the WSC content of mulberry silage. AVEO treatment significantly (*P* < 0.05) decreased the NH_3_-N content compared with that in the CK silage. Similar to the result of our study, cinnamon essential oil also reduced the NH_3_-N content in pea silage (Cantoia Júnior et al., 2020). This may have occurred because the essential oil could inhibit the growth of NH_3_-N-producing bacteria, thereby reducing the NH_3_-N content of silage (Tang et al., 2021).

### Fermentation characteristics of paper mulberry silages

The fermentation quality of the PM silages is shown in Table 2. The FA- and AVEO-treated silages exhibited lower (*P* < 0.05) pH values than the CK silage during the ensiling process. This may be attributable to the acidification function of FA (Wei et al., 2021). Administration of AVEO significantly increased the abundance of *Lactiplantibacillus* and decreased the abundance of *Proteobacteria*, such as *Desulfovibrio* and *Helicobacter*, which could be an explanation for the observed effects in this study (Chen et al., 2018). However, in our study, there was no difference in pH between the LAB- and CK-treated silages. A similar situation was reported by Cheng et al. (2021), who found that PM silage treated with a commercial inoculant (*Lactiplantibacillus*) did not exhibit improved fermentation quality. At later stages of fermentation, the FA-treated silage exhibited higher LA content and lower AA content than the CK silage (*P* < 0.05). However, the opposite results were obtained by Tyrolová et al. (2017) and Wei et al. (2021), who reported that FA treatment significantly (*P* < 0.05) decreased the LA content and increased the AA content of silage. This may be due to the differences in forage characteristics and in the FA application rate, which can lead to inconsistent results in the fermentation quality of silage (Huhtanen et al., 2013). However, after 60 days of ensiling, the LAB-treated silage exhibited lower LA content than the FA- and AVEO-treated silages but had the highest AA content (3.83% DM) (*P* < 0.05). This was similar to the results of Queiroz et al. (2013), who shown that LAB treatment increased the AA content but not the LA content in corn silage. A study from Driehuis and van Wikselaar (2000) demonstrated that changes in the LA and AA levels of silage were related to the pH and LAB types. The reason for the higher AA in the LAB-treated silage could be due to the ability of the added *L. buchneri* to convert LA to AA (Danner et al., 2003). After 60 days of ensiling, the AVEO-treated silage exhibited the highest (*P* < 0.05) LA content (6.47% DM) compared with the other silages. According to Vousough et al. (2009), mint essential oil has a stimulatory effect on the growth of several strains of *Lactiplantibacillus in vitro*. Therefore, the increase in LA content was attributed to AVEO significantly increasing the abundance of *Lactiplantibacillus* and decreasing the abundance of harmful microorganisms to metabolize LA (Chen et al., 2018). These results showed that AVEO could improve the fermentation quality of PM silage. Interestingly, PA and BA were not detected in any of the silages, which was similar to the results of Cheng et al. (2021), who found that neither PA nor BA was detected in PM silage treated with additives.

**TABLE 2:** Fermentation quality of paper mulberry silage.

Items	Treatment (T)	Ensiling days (D)					SEM	*P*-value		
		Day 3	Day 7	Day 15	Day 30	Day 60		T	D	T × D
pH	CK	5.77Aa	5.51Ab	5.45Abc	5.32Acd	5.25Ad				
	FA	4.22Cab	4.16Cab	4.02Cbc	3.85Cd	4.41Ba	0.011	<0.001	<0.001	<0.001
	LAB	5.70Aa	5.42Ac	5.31Ad	5.40Acd	5.55Ab				
	AVEO	4.59Ba	4.50Bab	4.47Bbc	4.36Bc	4.45Bbc				
LA (%DM)	CK	4.49ABbc	5.84Bab	6.55Aa	3.14Cc	1.30Cd				
	FA	3.70Bc	5.44Bb	3.15Bd	7.71ABa	4.62Bbc	0.102	<0.001	<0.001	<0.001
	LAB	1.92Cb	2.86Cb	3.16Aa	2.26Db	2.20Cb				
	AVEO	5.55Ac	6.02Ab	4.44Bc	7.56Aa	6.47Ab				
AA (%DM)	CK	0.77Bc	0.77Cc	1.09Bbc	1.54Bb	2.25Ca				
	FA	0.09Cd	3.10Aa	0.16Cba	1.36Bb	1.39Db	0.042	<0.001	<0.001	<0.001
	LAB	1.36Ac	2.69ABb	1.29Bc	2.35Ab	3.83Aa				
	AVEO	1.41Ad	2.10Bc	2.72Aab	2.35Abc	3.21Ba				
PA (%DM)	CK	ND	ND	ND	ND	ND				
	FA	ND	ND	ND	ND	ND	–	–	–	–
	LAB	ND	ND	ND	ND	ND				
	AVEO	ND	ND	ND	ND	ND				
BA (%DM)	CK	ND	ND	ND	ND	ND				
	FA	ND	ND	ND	ND	ND	–	–	–	–
	LAB	ND	ND	ND	ND	ND				
	AVEO	ND	ND	ND	ND	ND				

*^A–D^Means of additives treatments within a column with different superscripts differ on the same ensiling days (P < 0.05). ^a–d^Means of ensiling days within a row with different superscripts differ in the same additive treatment (P < 0.05). CK, without additives; FA, formic acid; LAB, Lactiplantibacillus plantarum combined with Lactiplantibacillus buchneri; AVEO, Amomum villosum essential oil; DM, dry matter; LA, lactic acid; AA, acetic acid; PA, propionic acid; BA, butyric acid; ND, not detected; T, treatment; D, ensiling days; T × D, interaction of treatments and ensiling days; SEM, standard error of the mean.*

### Bacterial community composition of paper mulberry silages

The bacterial diversity of the PM silages is shown in Table 3. The coverage value of all samples was more than 0.99, indicating that the sequencing depth was sufficient for determining the microbial composition. During the ensiling process, numerous microorganisms were replaced by an anaerobic LAB population, and the observed species index decreased, leading to the production of well-fermented silage. During ensiling, a decrease in the observed species index was observed in the AVEO-treated silage compared with the CK silage (*P* < 0.05). Similar results were found in the study of Dong et al. (2020), who reported that the observed species index in PM silage decreased after ensiling. The addition of AVEO during ensiling significantly decreased (*P* < 0.05) the bacterial alpha-diversity (ACE, Chao1, Shannon, and Simpson indexes) indexes of the PM silage compared to the CK silage. The results of our study are consistent with those of Dong et al. (2020), who showed that the Shannon, Simpson, and Chao1 indexes decreased after ensiling, suggesting that the diversity of the microbial community decreased when the growth of some microorganisms was inhibited by AVEO. However, in this study, there was no significant difference in bacterial alpha diversity among the silages treated with LAB, FA, and CK.

**TABLE 3:** Alpha diversity of bacteria of fresh and ensiled paper mulberry.

Item	Fresh	Treatment	Ensiling days (D)		SEM	*P*-value		
	forage	(T)	Day 3	Day 60		T	D	T × D
Observed species	162	CK	176AB	172AB				
		FA	190A	169A	1.627	<0.001	0.397	0.004
		LAB	171B	164B				
		AVEO	164C	144C				
ACE	180.88	CK	209.19A	201.56A				
		FA	207.48A	191.03A	1.879	0.003	0.087	0.055
		LAB	203.36A	187.84A				
		AVEO	187.75B	175.64B				
Chao1	181.22	CK	214.30A	203.00A				
		FA	208.75A	189.33A	2.490	0.006	0.154	0.149
		LAB	200.36A	188.09A				
		AVEO	185.65B	172.46B				
Simpson	0.32	CK	0.93AB	0.65AB				
		FA	0.88A	0.81A	0.017	0.005	<0.001	0.010
		LAB	0.93BC	0.50BC				
		AVEO	0.74C	0.57C				
Shannon	1.38	CK	4.90AB	2.95AB				
		FA	4.33A	4.15A	0.099	<0.001	<0.001	0.001
		LAB	4.91B	2.20B				
		AVEO	2.76C	2.06C				
Coverage	0.999	CK	0.997B	0.998B				
		FA	0.999B	0.998B	0.000	0.008	0.185	0.297
		LAB	0.998B	0.999B				
		AVEO	0.999A	0.999A				

*^A–C^ Means of additive treatments within a column with different superscripts differ on the same ensiling days (P < 0.05). CK, without additives; FA, formic acid; LAB, Lactiplantibacillus plantarum combined with Lactiplantibacillus buchneri; AVEO, Amomum villosum essential oil; T, treatment; D, ensiling days; T × D, interaction of treatments and ensiling days; SEM, standard error of the mean.*

**FIGURE 1:**
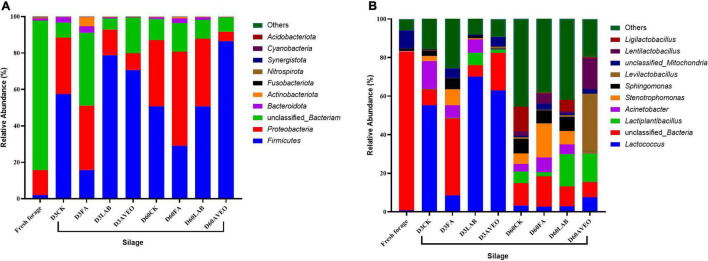
Relative abundance of the bacterial community at the phylum **(A)** and genus **(B)** levels in fresh paper mulberry and the control treatment without additives (CK) or with formic acid (FA); *Lactiplantibacillus plantarum* combined with *Lactiplantibacillus buchneri* (LAB); *Amomum villosum* essential oil (AVEO); D3, 3 days of ensiling; D60, 60 days of ensiling.

Changes in the bacterial community composition during the fermentation process in PM silages at the phylum level are shown in Figure 1A. *Firmicutes* and *Proteobacteria* were mainly detected during the ensiling process. The results of our study are consistent with those of He et al. (2021), who reported that *Firmicutes* and *Proteobacteria* were the main phyla detected during the ensiling process in PM silage. Moreover, the addition of LAB and AVEO reduced the relative abundance of *Proteobacteria* while increasing the relative abundance of *Firmicutes*. The addition of exogenous LAB (which are members of *Firmicutes*) increased the relative abundance of *Firmicutes* (Liu et al., 2019) and the acidic environment produced by LAB suppressed the growth of many undesirable microorganisms (Yan et al., 2019), resulting in a reduction in the relative abundance of *Proteobacteria*. Moreover, the main components of AVEO are alkenes, esters, and alcohols, and these chemicals have marked inhibitory effects on *Proteobacteria* (Knobloch et al., 1989; Zhang et al., 2011). Changes in the bacterial community composition during the fermentation process in PM silage at the genus level are shown in Figure 1B and Table 4. The main microorganisms in fresh PM were unculturable bacteria, which is consistent with the results of Cheng et al. (2021). Overall, both unclassified bacteria and *Lactococcus* were dominant in the PM silages at 3 days of ensiling. This result is consistent with those of Zhang et al. (2019), who found that the dominant genus in PM silage was *Lactococcus*. However, *Lactococcus* is considered an LA-producing microorganism in silage during the initiation of fermentation (Liu et al., 2019). Moreover, the abundance of *Lactococcus* in the LAB- and AVEO-treated silages increased (*P* < 0.05) compared to that in the CK treatment at 3 days of ensiling. This confirms our results for fermentation quality, with higher LA content observed in the LAB and AVEO treatments (Table 2) because the addition of LAB and AVEO inhibits the growth of Proteobacteria, such as *Desulfovibrio*, *Sutterella*, and *Helicobacter* (Chen et al., 2018; Dong et al., 2020), thus providing sufficient nutrients for the growth of *Lactococcus*. However, at 3 days of ensiling, the relative abundance of *Lactococcus* in FA-treated silage was lower (*P* < 0.05) than that in the CK treatment. This may be because FA rapidly reduces the pH and inhibits the growth of acid-intolerant microorganisms, such as *Lactococcus* (Dong et al., 2020). *Acinetobacter* was not detected in the AVEO-treated silage but could be detected in the other treated silages. We speculated that the level of *Acinetobacter* could not be determined due to the ability of AVEO to suppress bacterial growth. It has been found that *Acinetobacter* can utilize AA to survive in an anaerobic environment, and its abundance may increase with increasing AA content (Fuhs and Chen, 1975; Ogunade et al., 2017). Therefore, in our study, the abundance of *Acinetobacter* in the CK, LAB and FA treatments might have been related to the AA content. The relative abundance of *Lactococcus* decreased in all the silages treated for 60 days compared to 3 days due to a decrease in pH as the ensiling duration increased, thereby inhibiting the growth of acid-intolerant microorganisms (Dong et al., 2020). In addition, at 60 days of ensiling, the relative abundance of *Lactiplantibacillus* in the LAB- and AVEO-treated silages was higher than that in the CK silage. This is because the main component of the LAB additive was *Lactiplantibacillus*. The results of our study are consistent those of Chen et al. (2018), who found that administration of AVEO significantly increased the relative abundance of *Lactiplantibacillus.* Bornyl acetate is considered the main active ingredient of AVEO and exerts its antibacterial effect *via* modulation of p38 MAPK kinase and Caspase 3 expression (Chen et al., 2018). We found that *Levilactobacillus*, *Lentilactobacillus* and *Ligilactobacillus* were present in the PM silage after 60 days of ensiling. *Levilactobacillus* and *Lentilactobacillus* are heterofermentative LAB (Zheng et al., 2020). Species of *Ligilactobacillus* are homofermentative and express urease (Krumbeck et al., 2016). At 60 days of ensiling, the relative abundance of *Levilactobacillus* and *Lentilactobacillus* in the AVEO-treated silage was higher than that in the other silages. This may have been due to the inhibitory effects of AVEO on harmful bacteria making the relative abundance of LAB with strong acid tolerance higher than that in other silages. In summary, AVEO increased the abundance of *Lactococcus* in the early stage of ensiling and *Levilactobacillus* and *Lentilactobacillus* in the late stage of ensiling, resulting in improved PM silage quality.

**TABLE 4:** Changes in all dominant LAB in silages at the genus level.

Taxonomic level	Bacteria	Fresh forage	Ensiling days (D)	Treatments (T)				SEM	*P-value*		
				CK	FA	LAB	AVEO		T	D	T × D
Genus (%)	*Lactococcus*	0.72	Day 3	55.17A	8.54B	70.09A	62.94A	0.012	<0.001	<0.001	<0.001
			Day 60	3.35A	2.70B	2.82A	7.54A				
	*Lactiplantibacillus*	0.05	Day 3	0.17AB	0.19B	6.42A	1.64AB	0.013	0.090	0.019	0.569
			Day 60	5.90AB	2.06B	16.75A	14.73AB				
	*Levilactobacillus*	0.02	Day 3	0.04B	0.09B	0.01B	0.85A	0.016	0.022	0.048	0.031
			Day 60	0.93B	0.43B	0.86B	30.23A				
	*Lentilactobacillus*	0.01	Day 3	0.06	0.04	0.01	0.01	0.013	0.265	0.047	0.263
			Day 60	2.32	5.30	0.91	16.11				
	*Ligilactobacillus*	0.02	Day 3	0.03A	0.05B	0.03AB	0.02B	0.006	0.016	0.002	0.016
			Day 60	12.68A	0.40B	5.97AB	0.50B				

*^A–B^ Means of additive treatments within a row with different superscripts differ on the same ensiling days (P < 0.05). CK, without additives; FA, formic acid; LAB, Lactiplantibacillus plantarum combined with Lactiplantibacillus buchneri; AVEO, Amomum villosum essential oil; T, treatments; D, ensiling days; T × D, interaction of treatments and ensiling days; SEM, standard error of the mean.*

### Functional prediction for the bacterial community

**FIGURE 2:**
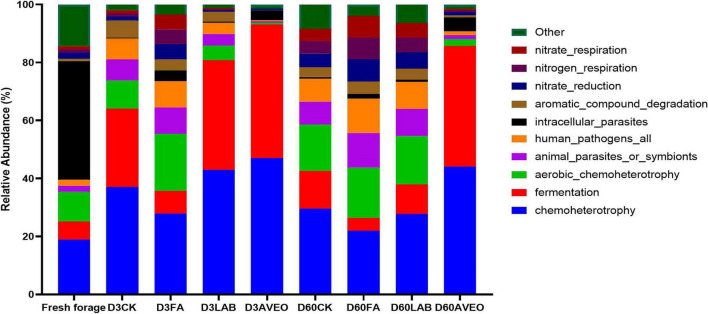
Functional prediction for the bacterial community in fresh paper mulberry and the control without additives (CK) or with formic acid (FA); *Lactiplantibacillus plantarum* combined with *Lactiplantibacillus buchneri* (LAB); *Amomum villosum* essential oil (AVEO); D3, 3 days of ensiling; D60, 60 days of ensiling.

The results of functional prediction for the bacterial community during silage fermentation are shown in Figure 2. Chemoheterotrophs were the primary functional components of the bacterial population in the silage, followed by fermenters, aerobic chemoheterotrophs, and animal parasites or symbionts. At 3 days of ensiling, treatments with AVEO and LAB promoted silage fermentation, whereas treatment with FA reduced the rate and extent of fermentation. The degree of fermentation was greater in the LAB and AVEO treatments due to the higher abundance of *Lactococcus*, which could be an explanation for the effects observed in this study. At 60 days of ensiling, treatment with AVEO promoted silage fermentation. Similar results were found in the study of Li et al. (2021b) who reported that treatment with an additive promoted the silage fermentation. Since the AVEO treatment showed increased abundances of LAB, such as *Levilactobacillus* and *Lentilactobacillus*, the degree of fermentation in the PM silage increased under AVEO treatment. Treatment with AVEO reduced the abundances of aerobic chemoheterotrophs and animal parasites or symbionts compared to the control. Furthermore, Tang et al. (2021) reported that an essential oil induced bacterial death by inhibiting the TCA cycle, decreasing ATP and ROS generation, and enhancing SOD activity, which resulted in a reduction in the abundance of aerobic bacteria and animal parasites.

## Conclusion

Additives can affect the fermentation quality of PM silage by altering the bacterial community structure during ensiling, with the AVEO additive providing the best effect. Mainly, AVEO increased the abundance of *Lactococcus* in the early stage of ensiling and *Levilactobacillus* and *Lentilactobacillus* in the late stage of ensiling, resulting in improved PM silage quality. Therefore, AVEO could potentially be developed as a new additive for improving the fermentation quality of silage.

## Data availability statement

The original contributions presented in the study are included in the article/supplementary material, further inquiries can be directed to the corresponding authors.

## Author contributions

All authors listed have made a substantial, direct, and intellectual contribution to the work, and approved it for publication.

## Conflict of interest

The authors declare that the research was conducted in the absence of any commercial or financial relationships that could be construed as a potential conflict of interest.

## Publisher’s note

All claims expressed in this article are solely those of the authors and do not necessarily represent those of their affiliated organizations, or those of the publisher, the editors and the reviewers. Any product that may be evaluated in this article, or claim that may be made by its manufacturer, is not guaranteed or endorsed by the publisher.

## Abbreviations

PM: paper mulberry
FA: formic acid
AVEO: *Amomum villosum* essential oil
LAB: lactic acid bacteria
OTU: operational taxonomic unit
PCR: polymerase chain reaction
DM: dry matter
CP: rude protein
NDF: neutral detergent fiber
ADF: acid detergent fiber
WSC: water-soluble carbohydrate
NH_3_-N: ammonia nitrogen
FM: fresh matter
LA: lactic acid
AA: acetic acid
PA: propionic acid
BA: butyric acid.
